# Whole-exome sequencing is a powerful approach for establishing the etiological diagnosis in patients with intellectual disability and microcephaly

**DOI:** 10.1186/s12920-016-0167-8

**Published:** 2016-02-04

**Authors:** Patrick Rump, Omid Jazayeri, Krista K. van Dijk-Bos, Lennart F. Johansson, Anthonie J. van Essen, Johanna B. G. M. Verheij, Hermine E. Veenstra-Knol, Egbert J. W. Redeker, Marcel M. A. M. Mannens, Morris A. Swertz, Behrooz Z. Alizadeh, Conny M. A. van Ravenswaaij-Arts, Richard J. Sinke, Birgit Sikkema-Raddatz

**Affiliations:** 1 Department of Genetics, University of Groningen, University Medical Centre Groningen, P.O. Box 30.001, 9700 RB Groningen, The Netherlands; 2 Department of Clinical Genetics, University of Amsterdam, Academic Medical Centre, Amsterdam, The Netherlands; 3 Department of Genetics, University of Groningen, University Medical Centre Groningen, Genomics Coordination Centre, Groningen, The Netherlands; 4 Department of Epidemiology, University of Groningen, University Medical Centre Groningen, Groningen, The Netherlands

**Keywords:** Autosomal recessive inheritance, *ASPM*, *BRCA2*, *CASK*, *DYRK1A*, *ERCC8*, *KIF11*, Microcephaly, *RAB3GAP1 RNASEH2B*, *RTTN*, Whole-exome sequencing

## Abstract

**Background:**

Clinical and genetic heterogeneity in monogenetic disorders represents a major diagnostic challenge. Although the presence of particular clinical features may aid in identifying a specific cause in some cases, the majority of patients remain undiagnosed.

Here, we investigated the utility of whole-exome sequencing as a diagnostic approach for establishing a molecular diagnosis in a highly heterogeneous group of patients with varied intellectual disability and microcephaly.

**Methods:**

Whole-exome sequencing was performed in 38 patients, including three sib-pairs, in addition to or in parallel with genetic analyses that were performed during the diagnostic work-up of the study participants.

**Results:**

In ten out of these 35 families (29 %), we found mutations in genes already known to be related to a disorder in which microcephaly is a main feature. Two unrelated patients had mutations in the *ASPM* gene. In seven other patients we found mutations in *RAB3GAP1*, *RNASEH2B, KIF11*, *ERCC8*, *CASK*, *DYRK1A* and *BRCA2*. In one of the sib-pairs, mutations were found in the *RTTN* gene. Mutations were present in seven out of our ten families with an established etiological diagnosis with recessive inheritance.

**Conclusions:**

We demonstrate that whole-exome sequencing is a powerful tool for the diagnostic evaluation of patients with highly heterogeneous neurodevelopmental disorders such as intellectual disability with microcephaly. Our results confirm that autosomal recessive disorders are highly prevalent among patients with microcephaly.

**Electronic supplementary material:**

The online version of this article (doi:10.1186/s12920-016-0167-8) contains supplementary material, which is available to authorized users.

## Background

Microcephaly, a disproportionally small head size defined as occipitofrontal circumference at or below −3 standard deviations (SD), is an important neurological sign usually associated with developmental delays and intellectual disability [1]. Microcephaly can be congenital or may develop later after birth. It can occur as a more or less isolated finding or as one of the features of a more complex syndrome. To date, according to the London Medical Database [2], more than 800 syndromes with microcephaly have been described, reflecting an enormous number of possible diagnoses. Microcephaly may stem from a wide variety of genetic or non-genetic conditions and is considered to be a consequence of abnormal brain development. Non-genetic causes include foetal alcohol exposure, perinatal infections, asphyxia or haemorrhage. Microcephaly can also occur in patients with inborn errors of metabolism, but this explains only a small proportion of the total cases [3]. In the majority of patients a genetic cause is suspected [4]. Chromosomal abnormalities may account for 15–20 % of patients [5]. In a retrospective study of 680 children with microcephaly [6], a specific genetic cause was detected in 15.3 % of them, with numerical chromosome aberrations accounting for 6.8 % and microdeletions/duplications and monogenetic disorders accounting for the other 8.5 %. Over the last few years the number of genes related to microcephaly has been rising, primarily as a result of the increased use of whole-exome sequencing (for an overview see [7]). This clinical and genetic heterogeneity represents a major diagnostic challenge. Although the presence of additional clinical findings may aid in identifying a specific cause in some cases, the majority of patients with microcephaly remain undiagnosed. Yet an early and specific diagnosis is important because it can provide relevant information about disease prognosis, appropriate medical or supportive care, reproductive consequences for parents and other family members, and prenatal diagnostic options. It may also preclude unnecessary further, and possibly invasive, diagnostic tests and evaluations.

The diagnostic work-up of microcephaly is extensive and includes brain imaging, metabolic and ophthalmologic evaluations, skin or muscle biopsies, karyotyping or microarray analysis, and mutation analyses of microcephaly genes. However, the use of targeted approaches, such as candidate gene testing by Sanger sequencing, is rather limited and their diagnostic yield is generally low. In contrast, because whole-exome sequencing does not require a priori knowledge of the gene or genes responsible for the disorder under investigation [8], it has been proven to be a very effective technique for identifying new genetic causes of microcephaly [9, 10].

In this study, we investigated the utility of whole-exome sequencing as a diagnostic approach for establishing a molecular diagnosis in a highly heterogeneous group of patients with microcephaly and intellectual disability.

## Methods

### Study subjects

We performed whole-exome sequencing in 38 patients, including three sib-pairs, with severe microcephaly and varied intellectual disabilities (Table 1). Patients were recruited, both prospectively and retrospectively, from the population of patients with intellectual disability referred to the genetics departments of the University Medical Centre Groningen and the Academic Medical Centre in Amsterdam. Informed consent was obtained from the participating families and the study protocol was approved by the Ethics Committee of the University Medial Centre Groningen.

**Table 1:** Characteristics of 38 patients with intellectual disability and microcephaly on whom whole-exome sequencing was performed

Patient	Age	Sex	OFC (Z-score)	Brain imaging (MRI)	Additional clinical features
1	8 years	f	−3.0	Thin corpus callosum, mild enlarged ventricles, hypoplasia of vermis and cerebellum	Bilateral congenital cataracts, microphthalmia, pale optic discs, hypogenitalism, axial hypotonia, hip dislocation, joint contractures, severe intellectual disability
2	5 years	f	−5.3	Mild enlarged ventricles	Intra-uterine growth retardation, neonatal hypoglycaemia, sensineural deafness, moderate intellectual disability
3	3 years	f	−6.3	Simplified gyral pattern, thin corpus callosum, wide peripheral liquor spaces	Feeding problems, recurrent respiratory infections, seizures, severe developmental delay
4	2 years	m	−4.6	Normal	Chorioretinopathy, mild developmental delay, hip dysplasia (mother with microcephaly and learning problems)
5	8 years	m	−3.3	Normal	Hypotelorism, cryptorchid testis, short stature, mild myopia, hyperactivity, intellectual disability
6	31 year	f	−5.0	NA	Mild intellectual disability, seizures, behavioural problems (brother with intellectual disability and microcephaly)
7	4 years	m	−3.6	Thin corpus callosum, delayed myelinisation, hypoplasia of frontal lobes	Strabismus, myoclonic jerks, spastic paraplegia, severe developmental delay
8(^a^)	5 years	f	−4.4	Diffuse pachygyria	Short stature, moderate intellectual disability, bilateral metatarsus primus varus
9(^a^)	10 year	f	−5.2	Mild frontal lissencephaly, posterior frontal pachygyria and parieto-occipital subcortical band heterotopia	Short stature, Tetralogy of Fallot, embryotoxon posterior, moderate intellectual disability (sister of patient 8)
10(^b^)	56 years	f	NA	Normal CT-scan	Severe microcephaly, chorioretinopathy, seizures, autism spectrum disorder, severe intellectual disability
11(^b^)	57 years	f	−7.1	NA	Chorioretinopathy, seizures, autism spectrum disorder, severe intellectual disability (sister of patient 10)
12	3 years	m	−4.6	Temporal and parieto-occipital atrophy	Cryptorchid testes, spasticity, feeding problems, nephrocalcinosis, seizures, severe developmental delay
13	10 year	m	−5.0	Simplified gyral pattern, mild cerebellar vermis hypoplasia	Hyperactivity, behavioural problems, speech delay, mild-moderate intellectual disability
14	1 year	m	−3.2	Megacisterna magna, prominent sulci of cerebellum	Brachycephaly, carp shaped mouth, uplifted ear lobes, mild hypotonia, developmental delay
15	0.5 years	f	−7.0	Simplified gyral pattern, sub-cortical areas with high signal intensity, prominent ventricles, small cerebellum	Infantile spasms, hyperkinesia, hypertonia, feeding problems, severe developmental delay
16	3 years	m	−3.3	Leucodystrophy, cerebral and cerebellar atrophy	Short stature, truncal hypotonia, spasticity, contractures, scoliosis, deafness, pigmentary retinopathy, mildly enlarged liver and elevated aminotransferase, feeding problems, cryptorchid testis, severe developmental delay, progressive disease course (consanguineous parents)
17	2 years	f	−3.0	NA	Short stature, embryotoxon posterior, feeding problems, developmental delay
18	23 years	m	−3.0	Hypoplasia of cerebral and cerebellum hemispheres, large cisterna magna, abnormal myelinisation	Seizures, hypotonia, thoracic kyphosis, camptodactyly, horseshoe kidney, small penis, auto mutilation, severe intellectual disability
19	22 years	f	−4.0	Atrophy, mild enlarged ventricles on CT-scan	Umbilical hernia, hip dysplasia, scoliosis, spasticity, feeding problems, severe intellectual disability, seizures, no speech, prominent upper incisors
20	4 years	m	−3.9	Normal	Metopic ridge, pancreas annulare, unilateral cleft lip and palate, short stature, severe developmental delay
21	11 year	f	−4.8	Hypoplasia frontal lobes, delay of myelinisation	Chorioretinopathy, mild cortical cataracts
22	5 years	f	−8.0	Delayed myelinisation, thin corpus callosum	Severe intellectual disability, hyperactivity disorder
23	2 years	m	−4.9	Wide peripheral liquor spaces	Low birth weight, brachycephaly, hypertonia, severe developmental delay (consanguineous parents)
24	19 years	f	−3.0	Focal temporal cortical dysplasia	Postnatal growth retardation, obesity, seizures, primary amenorrhea, hepatocellular carcinoma, mild-moderate intellectual disability with regression
25	2 years	m	−4.5	Normal	Short stature, adducted thumbs, hyperinsulinism, hypothyroidism, feeding problems, developmental delay
26	5 years	f	−3.9	Multiple intra cerebral calcifications on CT-scan	Feeding problems, short stature, seizures, spasticity, severe developmental delay
27(^c^)	5 years	m	−4.5	Diffuse polymicrogyria	Seizures, ventricle septum defect, feeding problems, spasticity, severe developmental delay (consanguineous parents)
28(^c^)	13 years	f	NA	Diffuse polymicrogyria	Severe microcephaly, seizures, spasticity, severe intellectual disability (sister of patient 27)
29	1 year	f	−3.0	Normal	Feeding problems, recurrent ear infections, vesicoureteral reflux, developmental delay (consanguineous parents)
30	1 year	f	−5.2	Simplified gyral pattern, hypoplasia of frontal lobes and corpus callosum	Low birth weight, mild developmental delay
31	3 years	m	−3.4	Normal	Microtia, iris and choroid coloboma, blepharophimosis, hypertelorism, micropenis, short stature, ventricle septum defect, inguinal hernia, feeding problems, developmental delay (consanguineous parents)
32	14 years	f	−6.8	Simplified gyral pattern	Seizures, behavioural problems, short stature, obesity, severe intellectual disability
33	2 years	f	−3.1	Mild delay of myelinisation, wide liquor spaces	Multicystic renal dysplasia, liver cysts and liver fibrosis, insulin-dependent diabetes, sick thyroid syndrome, hypertension, mixed hearing loss, developmental delay, mild complex III deficiency
34	0 year	f	−3.9	Abnormal gyration and myelinisation, small cerebellum	Intra-uterine growth retardation, maternal oligohydramnios, progressive renal failure (diffuse mesangial glomerulo sclerosis), beaked nose, large floppy ears, long fingers
35	4 years	m	−4.4	Possible polymicrogyria	Myopia, severe developmental delay
36	16 years	f	−3.7	Abnormal gyration parieto-temporal, hypoplasia posterior part of corpus callosum	Short stature, behavioural problems, mild mental retardation
37	2 years	m	−3.5	Small brain, no other abnormalities	Short stature, atrial septum defect, hypotonia, hypospadia, blepharophymosis, ear tag, developmental delay
38	0.5 years	m	−5.2	Wide peripheral liquor spaces, abnormal gyration fronto-parietal, small vermis, small pons	Peripheral hypertonia, axial hypotonia, seizures, feeding problems, developmental delay

(^a^), (^b^), (^c^) indicating the three sib-pairs

NA, not available

OFC, occipital frontal head circumference (expressed in standard deviations below the mean (Z-score) according

The clinical characteristics of all 38 patients are summarized in Table 1. The average age of the patients was 10 years (range 0 to 57 years). The majority (87 %) were children, with only five patients over 18 years of age. Severe microcephaly was defined as an occipital frontal head circumference of at least 3 SDs below the age-related mean according to Dutch national reference curves. The mean occipital frontal head circumference Z-score for our patients was −4.5 SD (range −3 to −8 SD). Consanguinity was reported for the parents of six patients (patients# 16, 23, 27, 28, 29, 35), including one of the three sib-pairs. Phenotype information was retrieved from medical records and provided by the referring physicians.

All patients had undergone genetic testing during their routine diagnostic work-up. In addition to whole-exome sequencing, the work-up may have included standard chromosome analysis, metabolic screening, or DNA sequencing of individual or multiple genes. On average, 2.8 DNA tests (range 0 to 9 tests) were performed per family (Additional file 1: Table S1). The most frequently tested gene was *ASPM*. This gene was analysed in seven (20 %) of the families.

### Microarray analysis

SNP array analysis was performed in all patients prior to inclusion using the IlluminaHumanCytoSNP-12 v2.1 DNA Analysis BeadChip following the manufacturer’s instructions (Illumina, San Diego, CA, USA). Normalized intensity and allelic ratios were analysed using GenomeStudio Data Analysis Software and the cnvPartition v3.1.6 algorithm to assess copy number variation. No causal copy number variants were found (data not shown). Homozygosity was determined as were, if possible, any shared haplotypes within a family. Shared homozygous stretches between the siblings with consanguineous parents were detected by selecting regions in which both siblings shared the same homozygous alleles; for the sib-pair with a recessive disorder we selected regions in which they shared the same combination of alleles, and for the sib-pair with a dominant disorder we selected regions in which they shared at least one allele at each SNP position. Stretches of homozygosity were detected within a single patient by selecting regions that only showed homozygous alleles.

### Whole-exome sequencing

Library preparation was based on the SureSelect All Exon V4 bait protocol (Agilent Technologies, Inc., Santa Clara, CA, USA), and carried out on a PerkinElmer® SciClone NGS workstation (PerkinElmer, Inc., Waltham, MA, USA). Briefly, 3 micrograms of genomic DNA were randomly fragmented with ultrasound using a Covaris® instrument (Covaris, Inc., Woburn, MA, USA). Adapters were ligated to both ends of the resulting fragments. Fragments with an average insert size of 220 bp were excised using the LabChip XT® gel system (PerkinElmer) and amplified by PCR (98^o^ C 2 min; 11cycli of 98 °C 30s, 65 °C 30s, 72 °C 1 min; 72 °C 10 min). The quality of the product was verified by capillary electrophoresis on the LabChip GX instrument (PerkinElmer). Of this product, 500 ng was subsequently hybridized to the Agilent SureSelect® All Exon V4 bait pool (Agilent Technologies, Inc.). During subsequent enrichment with PCR (98 °C 2 min; 11 cycli of 98 °C 30s, 57 °C 30s, 72 °C 1 min; 72 °C 10 min), individual samples were bar-coded and the quality of the product was again verified on the LabChip GX instrument. The product had an average fragment size of 360 bp, and was sequenced using 100 base pair reads paired-end on an Illumina® HiSeq2000 in pools of four samples per lane (Illumina). Resulting image files were processed including demultiplexing using standard Illumina® base calling software.

All lanes of sequence data were aligned to the human reference genome build b37, as released by the 1000 Genomes Project [11], using Burrows-Wheeler Aligner [12]. Subsequently the duplicate reads were marked. Using the Genome Analysis Toolkit (GATK) [13], re-alignment around insertions and deletions detected in the sequence data and in the 1000 Genomes Project pilot [11] was performed, followed by base quality score recalibration. During the full process the quality of the data was assessed by performing Picard [14], GATK Coverage and custom scripts. SNP and indel discovery was done using GATK Unified Genotyper and Pindel [15], respectively, followed by annotation using SnpEff [16]. This production pipeline was implemented using the MOLGENIS compute [17] platform for job generation, execution and monitoring. For each sample, a vcf file was generated that included all variants. Mean target coverage of 64x was achieved, with more than 82 % of targeted bases having at least 20x coverage (Additional file 2: Table S2). Sample swab was excluded by a concordance check with the SNP information from the array.

### Data interpretation

For data interpretation we uploaded the vcf files to Cartagenia NGS bench (Cartagenia, Leuven, Belgium) and then filtered the variants. We removed variants covered by less than five sequence reads and possibly benign variants annotated in dbSNP133, the 1000 Genomes project, the Seattle exome database [18], or the GoNL database [19] with an allele frequency above 2 %. Additionally, variants that were annotated in eight control samples, and for which exome sequencing was performed in the same sequence run, were also removed because they were considered to be artefacts. The remaining variants were filtered on any occurrence in known genes related to the phenotype, using a filter with selected phenotype traits [HPO-terms for microcephaly (HP:0011451, HP:0005484, HP:0000253), abnormalities of the nervous system (HP:0002011, HP:0000707), and abnormality of the head (HP:0000234)] [20] in the Cartagenia filter tree. The remaining variants were assumed to be related to the phenotype and further filtered on an inheritance model. First, an autosomal recessive inheritance model was applied for gene identification, then an autosomal dominant or X-linked inheritance model was applied. We considered variants resulting in transcriptional or splice site effects as potentially pathogenic. For the remaining group of variants, including those without a phenotypic match, we checked for their presence in the professional HGMD database (Biobase-international, Beverly, MA, USA) and manually checked the relationships between variants and possible phenotypes. The effect of potentially pathogenic variants was explored in the Alamut prediction program (Alamut, Rouen, France) or based on information from available databases and literature.

### Validation of mutations by Sanger sequencing

Candidate pathogenic variants were validated by Sanger sequencing. When available, we studied segregation of these confirmed variants by investigating DNA samples from additional family members. Sequencing analysis was carried out using flanking intronic primers (primer sequences are available upon request). The forward primer was designed with a PT1 tail (5′-TGTAAAACGACGGCCAGT-3′) and the reverse primer with a PT2 tail (5′-CAGGAAACAGCTATGACC-3′). PCR was performed in a total volume of 10 μl containing 5 μl AmpliTaq Gold ®Fast PCR Master Mix (Applied Biosystems, Foster City, CA, USA), 1.5 μl of each primer with a concentration of 0.5 pmol/μl (Eurogentec, Serian, Belgium) and 2 μl genomic DNA in a concentration of 40 ng/μl. Samples were PCR amplified and sequenced according to our standard diagnostic protocols (available upon request).

### Causality criteria

To designate candidate pathogenic variants as causative, variants confirmed by Sanger sequencing were required to occur in genes in which mutations are known to cause a disorder with clinical features consistent with the patient’s phenotype. All variants were either reported in the literature or HGMD database [21] to be deleterious, classified as truncating or having a splice site effect, or predicted to be deleterious at least in three prediction algorithms in Alamut. The presence of candidate pathogenic variants was checked in the 1000 Genomes project, the Seattle exome database [18], Exome Variant Server [22], and Exome Aggregation Consortium (ExAC) [23]. Furthermore, when possible, familial segregation was investigated to determine whether this was consistent with the expected mode of inheritance. Causative variants were then reported to the referring physicians.

## Results

On average 34,580 single-nucleotide variants and small insertions or deletions were found in each patient by whole-exome sequencing (see Additional file 3: Table S3). A molecular diagnosis could be established in 11 patients from 10 families, corresponding to a diagnostic yield of 29 %. The inheritance of these mutations were autosomal recessive (*N* = 7 families), autosomal dominant (*N* = 2) and X-linked (*N* = 1). In three patients, a molecular diagnosis was reached during their diagnostic work-up while performing exome sequencing (patient 1, patient 26 and patient 32). All three of these diagnoses were also reached by whole-exome sequencing. In the remaining 25 families, we identified five possibly candidate genes (data not shown). However, for none of these candidates a causal relationship to the phenotype of the patients could be proven.

Mutations were detected in nine different previously identified microcephaly genes: *ASPM*, *RAB3GAP1, RNASEH2B, KIF11, RTTN, ERCC8*, *CASK*, *DYRK1A* and *BRCA2* (Table 2)*.* None of these mutations was homo- or heterozygous present in the databases of benign variants. One exception was the variant in *RNASEH2B* [c.529G > A]. This mutation has been described before [24] and is predicted to be pathogenic by Alamut . It was heterozygous reported in the ExAC database with a frequency of 0.13 %.

**Table 2:** Established molecular diagnoses in patients with microcephaly using whole-exome sequencing

Patient	Gene	Transcript ID	Transcript variants	Protein variants	Zygosity	Related disorder	Inheritance	Reference for mutations
1	*RAB3GAP1*	NM_012233.2	c.475_478delACTG	p.T159Afs*19	Homozygous	Warburg Micro syndrome 1[MIM 600118]	AR	Aligianis et al., [26]
4	*KIF11*	NM_004523.3	c.1040dupT	p.S348Efs*8	Heterozygous	Microcephaly with or without chorioretinopathy, lymphedema, or mental retardation [MIM 152950]	AD	Current study
6	*ASPM*	NM_018136.4	c.7781_7784delAGAA c.3168 + 1G > C	p. K2595Yfs*20 p.?	Compound heterozygous	Primary microcephaly 5 [MIM 608716]	AR	Current study
8&9	*RTTN*	NM_173630.3	c.4186delC c.2594A > G	p.E1397Kfs*7 p.H865R	Compound heterozygous	Polymicrogyria with seizures [MIM 614833]	AR	Current study
16	*ERCC8*	NM_000082.3	c.295_297delinsTG	p.R99Sfs*26	Homozygous	Cockayne syndrome type A [MIM 216400]	AR	Current study
19	*CASK*	NM_003688.3	c.2302 + 2 T > G	p.?	Heterozygous	Mental retardation and microcephaly with pontine and cerebellar hypoplasia [MIM 300749]	XL	Current study
23	*DYRK1A*	NM_001396.3	c.1433delT	p.F478Sfs*114	Heterozygous	Autosomal dominant mental retardation 7 [MIM 614104]	AD	Current study
24	*BRCA2*	NM_000059.3	c.9672dupA	p.Tyr3225Ilefs*30	Homozygous	Fanconi anemia, complementation group D1 [MIM 605724]	AR	Tea et al., [42]
26	*RNASEH2B*	NM_024570.3	c.529G > A c.554 T > G	p.A177T p.V185G	Compound heterozygous	Aicardi-Goutières syndrome 2[MIM 610181]	AR	Crow et al., [24]
32	*ASPM*	NM_018136.4	c.2389C > T c.2053dupA	p.R797* p.N688Kfs*5	Compound heterozygous	Primary microcephaly 5 [MIM 608716]	AR	Passemard et al., [25] Current study

*AR* Autosomal recessive, *AD* Autosomal dominant, *XL* X-linked, *MIM* Mendelian inheritance in man number (http://omim.org)

p.? = the predicted effect on the protein is unknown

We identified *ASPM* mutations related to primary microcephaly type 5 [25] in two independent families. Patient 6 had two novel mutations in the *ASPM* gene: a deletion of four base pairs resulting in a frameshift and a splice site mutation. Sanger sequencing of the parents confirmed segregation. The other *ASPM* mutations (in patient 32) had already been detected by Sanger sequencing: a stop mutation and an insertion of one base pair resulting in a frameshift.

In two patients with clinically recognizable syndromes (i.e. Warburg Micro syndrome and Aicardi-Goutières syndrome), mutations in *RAB3GAP1* (patient 1) [26] and *RNASEH2B* (patient 26) [24] related to these conditions were identified during their diagnostic work-up as well as by whole-exome sequencing. In *RAP3GAP1* a homozygous deletion of four base pairs in exon 6 was detected. This deletion creates a frameshift starting at codon Thr159. The new reading frame ends in a stop codon 18 positions downstream.

A frame-shift mutation in the *KIF11* gene was identified in patient 4, who was known to have microcephaly and retinopathy, consistent with the phenotype. The mutation is an insertion of one base pair resulting in a frameshift and after eight amino acids causing a stop codon. The mutation in this patient proved to be inherited from the mother, who also had microcephaly and mild learning problems, but who was not found to have any ophthalmologic abnormalities. The unaffected maternal aunt of patient 4 did not carry the mutation.

We found a compound heterozygous frame-shift and missense mutation in the *RTTN* gene encoding the centrosome-associated protein Rotatin in one of the three sib-pairs (patients 8 and 9). The missense mutation was predicted as deleterious by several programs (including Polyphen, SIFT, and Mutation taster).

In the *ERCC8* gene a homozygous frame-shift mutation was found in patient 16, located in an 8 Mb homozygous region causing a stop codon further in the exon. Sanger sequencing of the parents confirmed that both were carriers of this heterozygous mutation.

We further found a splice-site mutation in the *CASK* gene in patient 19, and analysis of the parents confirmed that this was a *de novo* mutation. This mutation results in a predicted change of the splice site.

We identified a frame-shift mutation in the *DYRK1A* gene in patient 23, which was not inherited from the patient’s mother. Because DNA of the patient’s father was not available for testing, we were unable to confirm a *de novo* event in our patient, but the mutation is highly likely to be pathogenic.

A homozygous mutation in the *BRCA2* gene was detected in patient 24. Biallelic *BRCA2* mutations cause Fanconi anaemia complementation group D1 [27], a phenotype consistent with the phenotype of this patient. Sanger sequencing of the parents confirmed segregation. Since both parents of our patient were heterozygous for the mutation, and because of the associated increased risk for cancer, both of their families were offered appropriate genetic counselling and mutation screening. To date, no other family members have been reported to have cancer.

## Discussion

Overall, a molecular diagnosis was established in 29 % of the families with microcephaly studied using whole-exome sequencing. This diagnostic yield is comparable to the diagnostic yield of 25 % for whole-genome sequencing reported in a study of patients with a wide range of suspected genetic disorders, mostly related to neurologic conditions [28]. Our results are also in line with other studies on the diagnostic utility of whole-exome sequencing in genetically highly heterogeneous disorders. Whole-exome sequencing had a higher diagnostic yield than Sanger sequencing in patients with deafness (44 % vs. 10 %), blindness (52 % vs. 25 %), mitochondrial diseases (16 % vs. 11 %) and movement disorders (20 % vs. 5 %) [29]. In a study of 188 probands from families with consanguineous parents and two or more affected children, mutations in known genes were found in 27 % of the patients [8]. A similar diagnostic yield of approximately 25 % was obtained in a study on consanguineous families from Qatar [30].

We found mutations that are highly likely to be causative in nine different genes known to be associated with a disorder in which microcephaly is an important phenotypic feature*.* The *ASPM* gene is related to primary microcephaly type 5, which is the most common cause of autosomal recessive inherited primary microcephaly [31]. Although *ASPM* was the most frequently tested gene during the diagnostic work-up of the patients (Additional file 1: Table S1), in one of these two families this gene had not been tested. Mutations in the *KIF11* gene are known to cause autosomal dominant inherited microcephaly with or without chorioretinopathy, lymphedema, or mental retardation [32], a phenotype that is consistent with the clinical features of the patient in which we identified a *KIF11* mutation. Homozygous missense mutations in the *RTTN* gene have recently been identified in patients with microcephaly, intellectual disability, epilepsy and bilateral polymicrogyria [33]. The p. H865R mutation identified in our family results in a substitution of an amino acid that is located in the second Armadillo-like domain of Rotatin, a protein-protein interacting domain that is highly conserved among species. Sanger sequencing in both parents and two unaffected siblings confirmed that the mutations segregated with the clinical phenotype in this family, further proving causality. In our patients, the type of cortical malformations differed and the degree of microcephaly was more severe than in these published cases. It is known that such heterogeneity in distribution, severity and type of cortical malformations can occur in relation to mutations in one specific gene, as has been observed with mutations in the *GPR56* gene [33, 34]. *ERCC8* gene mutations cause Cockayne syndrome type A [35]. Although, in retrospect, this diagnosis is consistent with the clinical features of our patient, it had not been considered prior to our discovery of the *ERCC8* gene mutation by whole-exome sequencing. When the patient was re-evaluated, his facial features had changed substantially and had become more characteristic of Cockayne syndrome. Mutations in the *CASK* gene cause mental retardation with microcephaly and pontocerebellar hypoplasia [36]. This X-linked condition is seen more frequently in females than in males [37]. The presence of severe intellectual disability, spasticity, seizures, absence of speech, and prominent upper incisors reported in our *CASK* patient are known clinical features associated with this disorder [38]. Unfortunately, no brain MRI was available from this patient so it remains unknown whether she also had pontine or cerebellar hypoplasia. The absence of this information may also explain why this diagnosis had not been considered prior to whole-exome sequencing. *DYRK1A* mutations cause autosomal dominant mental retardation type 7, in which microcephaly is an important clinical finding [39, 40]. Typically, *de novo DYRK1A* mutations are found [39] as was most likely the case in our patient. Biallelic *BRCA2* mutations are known to cause Fanconi anaemia type D1 [41], and we found a homozygous mutation in this gene. Secondary microcephaly and hepatocellular carcinoma, as seen in our patient, are known features of Fanconi anaemia [27]. This particular *BRCA2* mutation has been identified in families with familial breast cancer [42].

In our cohort an autosomal recessive disorder was detected in 7/10 of the families with an established diagnosis, while only eight out of 35 (23 %) families had been suspected of having a recessive disorder (six consanguineous families and two families with affected siblings). This is consistent with the high empirical recurrence risk in sibs (approximately 15-20 %) of patients with severe primary microcephaly without an identified cause [43]. Our results suggest that autosomal recessive inheritance is far more frequent in patients with microcephaly than in patients with intellectual disability overall [28, 44]. In a study of 100 patients with severe intellectual disability, mostly *de novo* mutations with an assumed dominant effect were identified and only one patient was found to have an autosomal recessive disorder [45].

Our results confirm the clinical and genetic heterogeneity in patients with microcephaly. When whole-exome sequencing is performed early in the diagnostic work-up of patients, it may prevent other, unnecessary, diagnostic evaluations. Most of the patients in our study had had extensive diagnostic evaluations but these had not led to a specific diagnosis in the majority of cases. Neveling et al. calculated that when three or more genes per patient are investigated by Sanger sequencing, whole-exome sequencing is more cost effective and has a higher diagnostic yield [29].

## Conclusions

Our results demonstrate that whole-exome sequencing is a powerful tool for the diagnostic evaluation of highly heterogeneous neurodevelopmental disorders. We performed whole-exome sequencing in 35 families with intellectual disability and microcephaly. A molecular diagnosis could be established in 29 % of the studied families. In 70 % of the families an autosomal recessive condition was present, confirming that this inheritance mode is more prevalent among patients with intellectual disability who have microcephaly.

## Additional file

Additional file 1: Table S1. Genetic tests performed during the diagnostic work-up of the study participants. (DOC 55 kb)
Additional file 2: Table S2. Specification of the sequence data. (DOC 51 kb)
Additional file 3: Table S3. Overview of the number of variants detected at each filter step for each patient. (DOC 128 kb)

## Abbreviations

AD: autosomal dominant
AR: autosomal recessive
GATK: genome analysis toolkit
GoNL: genome of the netherlands
HGMD: human gene mutation database
HPO: human phenotype ontology
MIM: mendelian inheritance in man
NGS: next generation sequencing
PCR: polymerase chain reaction
SD: standard deviations
SNP: single nucleotide polymorphism
VCF: variant call format
WES: whole exome sequencing
XL: x-linked
