# Supplementary material for: Whole-exome sequencing is a powerful approach for establishing the etiological diagnosis in patients with intellectual disability and microcephaly
Source: BMC Med Genomics. 2016 Feb 4;9:7. doi: 10.1186/s12920-016-0167-8 (PMC4743197; doi:10.1186/s12920-016-0167-8)
Supplement: Additional file 1: Table S1. — Genetic tests performed during the diagnostic work-up of the study participants. (DOC 55 kb) [file 12920_2016_167_MOESM1_ESM.doc]

Supplemental table S1: Genetic tests performed during the diagnostic work-up of the study participants.

| **Patient** | **Number of tests** | **Genetic tests** |
| --- | --- | --- |
| 1 | 1 | *RAB3GAP* |
| 2 | 1 | *UBE3A* |
| 3 | 8 | *ASPM, NBN, MCPH1, WDR62, CDK5RAP2, CEP152, CENJP, STIL* |
| 4 | 0 | - |
| 5 | 5 | *SHH, SIX3, GLI2, ZIC2, TGIF* |
| 6 | 0 | - |
| 7 | 7 | *UBE3A*, *SNRPN* methylation, *MECP2, MCT8,* mtDNA (Mito chip), *TUBA1A, WDR62* |
| 8 | 0 | - |
| 9 | 7 | *DCX, LIS1,TUBA1A, FLNA, POMT1, POMGnT1,* mtDNA (m3243A>G; m8344A>G; m8993T>G/C) |
| 10 | 0 | - |
| 11 | 0 | - |
| 12 | 1 | *FMR1* |
| 13 | 3 | *MCPH1, ASPM, FMR1* |
| 14 | 3 | *ZEB2, FMR1, MCT8* |
| 15 | 0 | - |
| 16 | 6 | *FMR1, MCT8, POLG, TIMM8A, ECGF1(MNGIE),* mtDNA (Affymetrix resequencing array 2.0) |
| 17 | 0 | - |
| 18 | 1 | *MECP2* |
| 19 | 2 | *MECP2, CDKL5* |
| 20 | 1 | *MYCN* |
| 21 | 1 | Leber Congenital Amaurosis chip (Asper version 2010) |
| 22 | 7 | *WDR62, CDK5RAP2, CENPJ, CEP152, STIL, MCPH1, ASPM* |
| 23 | 0 | - |
| 24 | 3 | *CDKL5, MECP2, FMR1* |
| 25 | 7 | *KCNJ11, ABCC8, GCK*, *H19/LIT1* methylation, *GHR, STAT5B, IGF1* |
| 26 | 9 | *MECP2, CDKL5, UBE3A, TCF4, FOXG1, RNASEH2A, RNASEH2B, RNASEH2C, TREX1* |
| 27 | 1 | *GPR56* |
| 28 | 0 | - |
| 29 | 0 | - |
| 30 | 2 | *ASPM, MCPH1* |
| 31 | 4 | *CHD7, KAT6B, MED12, UBE3B* |
| 32 | 2 | *ASPM, MCPH1* |
| 33 | 6 | *HNF1β, PKHD1, NPHP3*, Bardet-Biedl syndrome micro-array (Asper version 5), mtDNA (Mito chip), *BCS1L* |
| 34 | 0 | - |
| 35 | 4 | *MCPH1, ASPM, NBS*, Fanconi anemia (mitomycine C test) |
| 36 | 3 | *ASPM, STIL, MCPH1* |
| 37 | 0 | - |
| 38 | 3 | *ASPM, STIL, MCPH1* |
