# Supplementary material for: Whole-exome sequencing is a powerful approach for establishing the etiological diagnosis in patients with intellectual disability and microcephaly
Source: BMC Med Genomics. 2016 Feb 4;9:7. doi: 10.1186/s12920-016-0167-8 (PMC4743197; doi:10.1186/s12920-016-0167-8)
Supplement: Additional file 2: Table S2. — Specification of the sequence data. (DOC 51 kb) [file 12920_2016_167_MOESM2_ESM.doc]

Supplemental table S2: Specification of the sequence data

| Samples | Mean | Min | Max |
| --- | --- | --- | --- |
| Genome size (bp) | 3101804739 | 3101804739 | 3101804739 |
| Bait size (bp) | 51695938 | 51374954 | 51756122 |
| Target size (bp) | 51695938 | 51374954 | 51756122 |
| Total number of reads | 119523589 | 75682208 | 174102599 |
| On target bases (Mb) | 3306.90 | 2141.65 | 4660.77 |
| Aligned bases (Mb) | 8863.88 | 5714.48 | 12854.38 |
| Mean bait coverage | 62.51 | 40.11 | 87.64 |
| Mean target coverage | 63.97 | 41.38 | 90.05 |
| Fraction bp on bait | 0.28 | 0.20 | 0.37 |
| Fraction bp near bait | 0.12 | 0.08 | 0.22 |
| Fraction bp off bait | 0.38 | 0.26 | 0.49 |
| Fraction bp not aligned | 0.11 | 0.05 | 0.16 |
| Capture specificity | 0.62 | 0.51 | 0.74 |
| Fraction target covered ≥ 2x | 0.96 | 0.95 | 0.99 |
| Fraction target covered≥ 10x | 0.90 | 0.84 | 0.96 |
| Fraction target covered ≥ 20x | 0.82 | 0.68 | 0.88 |
| Fraction target covered ≥ 30x | 0.71 | 0.52 | 0.83 |
| Fraction usable bases on target | 0.28 | 0.20 | 0.37 |
| Mean read length | 95 | 95 | 95 |
| Strand balance | 0.50 | 0.5 | 0.5 |
| Median insert size | 224 | 175 | 369 |
| Mean insert size | 226.49 | 172.72 | 353.71 |
| Standard deviation insert size | 52.22 | 25.41 | 99.69 |
| Number of SNPs for concordance | 2635 | 2103 | 3311 |
| Concordance | 1 | 1 | 1 |
|  |  |  |  |
|  |  |  |  |
| Name of the bait set(s) used in the hybrid selection for this project: | | |  |
| SureSelect All Exon 50MB baits hg19 human g1k v37 | |  |  |
