# Supplementary material for: Whole-exome sequencing is a powerful approach for establishing the etiological diagnosis in patients with intellectual disability and microcephaly
Source: BMC Med Genomics. 2016 Feb 4;9:7. doi: 10.1186/s12920-016-0167-8 (PMC4743197; doi:10.1186/s12920-016-0167-8)
Supplement: Additional file 3: Table S3. — Overview of the number of variants detected at each filter step for each patient. (DOC 128 kb) [file 12920_2016_167_MOESM3_ESM.doc]

Supplemental Table S3: Overview of the number of variants detected at each filter step for each patient

| Patient number | Input variants | Benign variant | Reference  (8 controls) | Phenotype  match | Transcriptional and/or Splice site effect | Homozygous | Compound variation | Mutation/  Gene | Phenotype  No match | Transcriptional and/or Splice site effect | Zygosity | Compound variation |
| --- | --- | --- | --- | --- | --- | --- | --- | --- | --- | --- | --- | --- |
| 1 | 30928 | 17946 | 2318 | 264 | 120 | 7* | 27 | *RAB3GAP1* | 2054 | 881 | 68 | 184 |
| 2 | 36294 | 20557 | 4210 | 476 | 181 | 38 | 19 | *No* | 3734 | 1367 | 272 | 262 |
| 3 | 34338 | 19709 | 1791 | 224 | 97 | 15 | 8 | *No* | 1567 | 673 | 40 | 81 |
| 4 | 34219 | 19809 | 1914 | 201 | 88* | 5 | 9 | *KIF11* | 1713 | 777 | 44 | 123 |
| 5 | 40079 | 23015 | 4923 | 552 | 215 | 8 | 53 | *No* | 4371 | 1799 | 84 | 425 |
| 6 # | 34137 | 19976 | 2003 | 212 | 105* | 3 | 20 | *ASPM* | 1791 | 789 | 41 | 136 |
| 7 | 34965 | 20321 | 1966 | 217 | 97 | 7 | 13 | *No* | 1749 | 799 | 39 | 136 |
| 8 | 36419 | 21686 | 2618 | 228 | 109 | 4 | 34* | *RTTN* | 2390 | 1083 | 35 | 293 |
| 9 | 34291 | 19600 | 1998 | 210 | 78 | 1 | 12* | *RTTN* | 1788 | 768 | 34 | 114 |
| 8 and 9 Common | 26227 | 16043 | 704 | 83 | 31 | 0 | 2* | *RTTN* | 607 | 304 | 2 | 24 |
| 10 | 32614 | 19045 | 1806 | 193 | 99 | 4 | 10 |  | 1613 | 741 | 17 | 106 |
| 11 | 35628 | 21231 | 3188 | 327 | 140 | 10 | 29 |  | 2861 | 1242 | 161 | 296 |
| 10 and 11 Common | 19845 | 12398 | 596 | 77 | 37 | 1 | 2 |  | 519 | 242 | 1 | 20 |
| 12 | 33353 | 19341 | 1826 | 201 | 87 | 1 | 10 | *No* | 1625 | 708 | 37 | 93 |
| 13 | 35439 | 20425 | 2692 | 317 | 123 | 11 | 10 | *No* | 2375 | 966 | 155 | 124 |
| 14 | 35192 | 20883 | 2241 | 233 | 117 | 9 | 20 | *No* | 2008 | 894 | 52 | 177 |
| 15 | 34349 | 20046 | 1818 | 232 | 114 | 2 | 17 | *No* | 1586 | 724 | 29 | 114 |
| 16 | 32762 | 18891 | 2135 | 229 | 116 | 6* | 21 | *ERCC8* | 1906 | 845 | 41 | 136 |
| 17 | 37165 | 22062 | 3738 | 354 | 158 | 7 | 33 | *No* | 3384 | 1484 | 150 | 470 |
| 18 | 29789 | 17311 | 1612 | 161 | 74 | 5 | 11 | *No* | 1451 | 629 | 41 | 130 |
| 19 | 31112 | 17278 | 3196 | 398 | 162* | 25 | 29 | *CASK* | 2798 | 1011 | 173 | 139 |
| 20 | 30992 | 17650 | 1671 | 174 | 86 | 2 | 16 | *No* | 1497 | 632 | 25 | 111 |
| 21 | 35253 | 20418 | 1817 | 222 | 101 | 5 | 15 | *No* | 1595 | 713 | 19 | 80 |
| 22 | 34002 | 19608 | 1823 | 218 | 113 | 7 | 23 | *No* | 1605 | 679 | 50 | 79 |
| 23 | 35081 | 20121 | 1823 | 201 | 80 | 3 | 12 | *DYRK1A* | 1622 | 732* | 55 | 98 |
| 24 | 34193 | 19909 | 1866 | 209 | 100 | 4* | 7 | *BRCA2* | 1657 | 776 | 37 | 106 |
| 25 | 35655 | 20697 | 2756 | 312 | 133 | 11 | 16 | *No* | 2444 | 1021 | 177 | 121 |
| 26 | 33854 | 19442 | 1916 | 206 | 108 | 3 | 18* | *RNASEH2B* | 1710 | 710 | 39 | 118 |
| 27 | 34252 | 19977 | 2354 | 249 | 114 | 12 | 20 |  | 2105 | 875 | 56 | 124 |
| 28 | 34825 | 20300 | 2492 | 268 | 116 | 7 | 19 |  | 2224 | 933 | 62 | 156 |
| 27 and 28 Common | 22983 | 14427 | 1115 | 124 | 55 | 3 | 11 |  | 991 | 440 | 18 | 52 |
| 29 | 34851 | 20262 | 1871 | 211 | 103 | 4 | 20 | *No* | 1660 | 734 | 41 | 68 |
| 30 | 34084 | 19709 | 1832 | 206 | 107 | 3 | 23 | *No* | 1626 | 724 | 18 | 105 |
| 31 | 33856 | 19832 | 1855 | 211 | 98 | 6 | 7 | *No* | 1644 | 744 | 51 | 132 |
| 32 | 34220 | 19823 | 1769 | 208 | 112 | 3 | 12* | *ASPM* | 1561 | 673 | 27 | 82 |
| 33 | 33778 | 19631 | 1703 | 200 | 93 | 2 | 14 | *No* | 1503 | 690 | 41 | 56 |
| 34 | 36031 | 20471 | 4002 | 423 | 146 | 24 | 24 | *No* | 3579 | 1259 | 267 | 209 |
| 35 | 37700 | 21522 | 4553 | 513 | 191 | 24 | 29 | *No* | 4040 | 1383 | 275 | 238 |
| 36 | 36388 | 21068 | 4551 | 486 | 185 | 34 | 30 | *No* | 4065 | 1477 | 268 | 304 |
| 37 | 36558 | 20657 | 4038 | 449 | 160 | 23 | 20 | *No* | 3589 | 1269 | 271 | 169 |
| 38 | 36067 | 20487 | 4055 | 429 | 160 | 27 | 31 | *No* | 3626 | 1357 | 277 | 268 |
| Mean of all Samples | 34597.71 | 20018.84 | 2545.79 | 279.58 | 120.89 | 10.14 | 19.56 | *10 cases solved* | 2266.21 | 941.32 | 93,92 | 162,18 |

| Gray rows represent sibs.  Common means common variants between sibs | | | |  |
| --- | --- | --- | --- | --- |
|  |  | |  | |
| # this patient has 1 splice site mutation and 1 deletion. Not found in the compound group. | | | | |
| * mutation found in this group |  |  | | |
